# Metabolomic Analysis of Plasma from Breast Cancer Patients Using Ultra-High-Performance Liquid Chromatography Coupled with Mass Spectrometry: An Untargeted Study

**DOI:** 10.3390/metabo12050447

**Published:** 2022-05-17

**Authors:** Patricia A. Da Cunha, Diana Nitusca, Luisa Matos Do Canto, Rency S. Varghese, Habtom W. Ressom, Shawna Willey, Catalin Marian, Bassem R. Haddad

**Affiliations:** 1 Lombardi Comprehensive Cancer Center and Department of Oncology, Georgetown University Medical Center, Georgetown University, Washington, DC 20057, USA; patricia311088@gmail.com (P.A.D.C.); luisaaamatos@gmail.com (L.M.D.C.); rency.varghese@georgetown.edu (R.S.V.); hwr@georgetown.edu (H.W.R.); shawna.willey@inova.org (S.W.); 2 Department of Biochemistry and Pharmacology, Victor Babeş University of Medicine and Pharmacy, Pta Eftimie Murgu Nr. 2, 300041 Timişoara, Romania; nitusca.diana@umft.ro (D.N.); cmarian@umft.ro (C.M.); 3 Center for Complex Networks Science, Victor Babeş University of Medicine and Pharmacy, Pta Eftimie Murgu Nr. 2, 300041 Timişoara, Romania; 4 Department of Surgery, Georgetown University Medical Center, Georgetown University, Washington, DC 20007, USA

**Keywords:** breast cancer, metabolomics, biomarkers, diagnostic, UHPLC/Q-TOF-MS

## Abstract

Breast cancer (BC) is one of the leading causes of cancer mortality in women worldwide, and therefore, novel biomarkers for early disease detection are critically needed. We performed herein an untargeted plasma metabolomic profiling of 55 BC patients and 55 healthy controls (HC) using ultra-high performance liquid chromatography coupled with quadrupole time-of-flight mass spectrometry (UHPLC/Q-TOF-MS). Pre-processed data revealed 2494 ions in total. Data matrices’ paired *t*-tests revealed 792 ions (both positive and negative) which presented statistically significant changes (FDR < 0.05) in intensity levels between cases versus controls. Metabolites identified with putative names via MetaboQuest using MS/MS and mass-based approaches included amino acid esters (i.e., N-stearoyl tryptophan, L-arginine ethyl ester), dipeptides (ile-ser, met-his), nitrogenous bases (i.e., uracil derivatives), lipid metabolism-derived molecules (caproleic acid), and exogenous compounds from plants, drugs, or dietary supplements. LASSO regression selected 16 metabolites after several variables (TNM Stage, Grade, smoking status, menopausal status, and race) were adjusted. A predictive conditional logistic regression model on the 16 LASSO selected ions provided a high diagnostic performance with an area-under-the-curve (AUC) value of 0.9729 (95% CI 0.96–0.98) on all 55 samples. This study proves that BC possesses a specific metabolic signature that could be exploited as a novel metabolomics-based approach for BC detection and characterization. Future studies of large-scale cohorts are needed to validate these findings.

## 1. Introduction

Breast cancer (BC) is the most frequently diagnosed and heterogeneous malignancies among women worldwide. It accounted for an estimated 276,480 new cases and 42,690 deaths occurring in 2020 in the US alone [1]. Being highly heterogeneous in both clinical presentation and outcomes, BC is classified and diagnosed based on the different molecular subtypes (estrogen and progesterone, hormone-based targeted classification, HER status), clinical manifestations, and treatment responses [2]. Early diagnosis using traditional screening techniques such as mammography and ultrasound has proven to significantly decrease mortality and improve overall survival [3,4]. However, although these screening methods are effective, all of them possess limitations from different perspectives, such as exposure to ionizing radiation, invasiveness, low specificity, and/or sensitivity [5]. In addition, despite all advances in current screening techniques, mortality rates are still elevated; therefore, there is an urgent need for novel, minimally-invasive detection methods, with improved sensitivity and specificity, in order to improve early diagnosis of BC.

Cancer cells possess unique features of the metabolic status that aid in the reorganization of metabolic networks involved in the adaptation to sustain cell growth and survival, the remodeling of tissues, and metastasis [6]. Warburg first discovered the “aerobic glycolysis” phenomenon in cancer cells, which leads to avoiding the unnecessary catabolic oxidation of nutrients and promotes anabolism for proliferating cells [7,8]. Ever since, this particular feature of cancer has been extensively exploited in research, showing that the dynamic portrait of the metabolic pattern of individuals offers new insights into a better understanding of the molecular changes, even in the most heterogeneous cancers, such as BC [9].

In this regard, metabolomics is the quantitative study of the complete metabolic profile from a biological sample, that reflects the ongoing cellular processes, and it is part of the large-scale “omics” approaches. In cancer, this high-throughput measurement technology has a great advantage and ability to provide a global snapshot of tumor biology, that includes development and progression, which could have the potential to enrich the common gold-standard, imaging-focused techniques, and provide novel comprehensions of carcinogenesis [10]. The quantification of metabolites in circulating samples such as blood-derived specimens is a minimally-invasive technique that can provide real-time results of the metabolomic profile of an individual with a certain disease or with cancer, and the comparison of the results with the metabolome of a disease-free control can bring valuable insights regarding the altered signaling pathways involved in the pathophysiology of a specific disease/tumor biology of cancer. The downstream-affected metabolites (with significant changes in concentrations between cases and controls) have a great potential to be used as future non-invasive biomarkers for a correct and early diagnosis. Such metabolomic fingerprinting techniques have already been studied in Chronic Obstructive Lung Diseases (COLD) and in various auto-immune diseases (i.e., rheumatoid arthritis, vitiligo, inflammatory bowel disease) [11,12]. However, metabolomics is merely a research lab-based technique, since the technological and computational limitations make the clinical practice translation a difficult task [13].

Mounting evidence from recent years proved that the metabolic profiling approach can be used in a growing number of applications for BC screening in particular, such as early disease detection, novel biomarker discovery, the monitoring of disease progression and occurrence, as well as analysis of altered metabolic pathways. A high number of studies demonstrated that metabolomics is an efficient tool for differentiating BC patients’ samples from normal ones [14,15,16,17]. Of interest, Dougan et al. (2018) investigated the metabolomic profile from pre-diagnostic plasma samples of 45 patients diagnosed with BC and 45 controls, using an ultra-high-performance liquid chromatography/electrospray ionization tandem mass spectrometry (UHPLC/MS) with an additional gas chromatography-mass spectrometry (GC/MS) platform, and found a statistically significant case-control difference (greater than 20%) in 24 metabolites [18]. In addition, Shen et al. (2013) previously reported a statistically significant difference in 117 plasma metabolites levels of African American women, when compared to Caucasian American women, and a significant case-control difference in 78 metabolites [17].

Interestingly, studies showed that metabolites levels from different cancer types could present a tissue-specific pattern, being, in general, unique for each type of cancer, and thus providing a new classification tool that can distinguish tumors in a tissue-of-origin manner [19]. To verify the discrimination power of metabolomics within different BC stages, Jobard et al. (2014) conducted a proton nuclear magnetic resonance (1H-NMR)-based metabolic profiling study and found 9 metabolites that could differentiate between metastatic BC and localized early disease, although a clear separation of metabolomic-based molecular subtype classification is still disputable to a certain extent [20,21].

In general, cancer cells produce tumor markers such as single-proteins, RNAs (microRNAs, lncRNAs, etc.), DNA, or even full metabolic signatures comprising multiple components [22,23,24]. Herein, we report an untargeted metabolomic analysis study that assesses potential differences in metabolites levels from 55 plasma samples of BC patients, compared to 55 plasma samples obtained from healthy controls (HC), in order to investigate the potential of a metabolic profiling approach as a minimally-invasive, liquid biopsy technique, that might open novel avenues for future applications of BC diagnosis and characterization.

## 2. Results

Demographic, clinical, and tumor characteristics of the 55 BC patients included in our study are presented in Table 1.

Regarding demographics, the mean age of the patients was 53 years old, with the vast majority being in the post-menopausal status (64%), and Caucasians (60%). The great majority of women never smoked (78%), with only 13% being current smokers at the blood collection period. Concerning tumors, the great majority were the invasive ductal carcinoma type (IDC, 64%), in early stages (0 + I + II, 81%), 72% having no lymph nodes, estrogen and progesterone receptors were positive (82% ER+, and 60% PR+), and HER2 was negative (71%).

After pre-processing the UHPLC-QTOF data, we identified 2494 ions, of which 1930 were in the positive mode, and 564 in the negative mode (Table 2).

**Figure 1:**
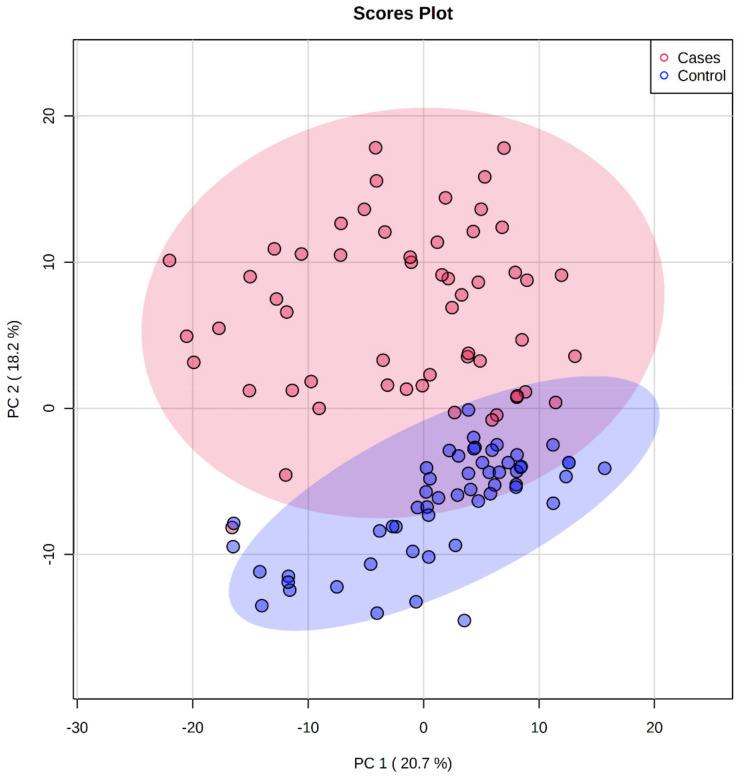
PCA 2D scores plot showing the first two principal components depicting the variance (shown in parenthesis) among the 55 samples.

**Figure 2:**
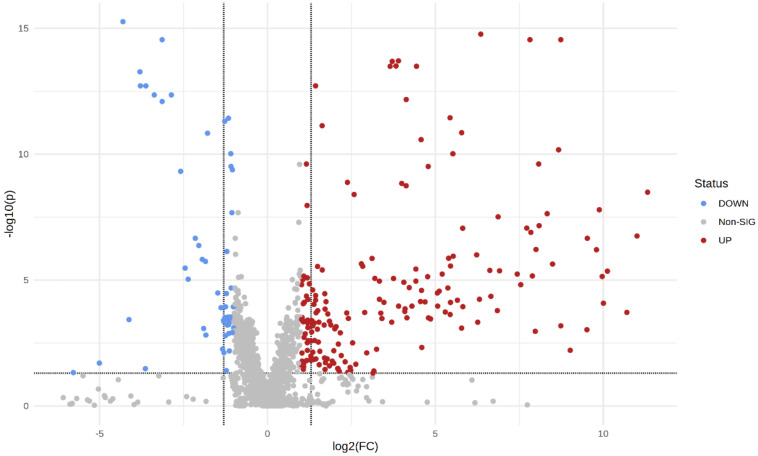
Important features selected by volcano plot with false discovery rate (FDR) < 0.05 and |FC| > 2 from univariate analysis. Red-colored dots denote up-regulated, blue-colored dots denote down-regulated, and grey-colored dots denote metabolites with non-significant change.

The generated data matrices were further used to select only the ions with statistically significant changes between plasma samples from patients with BC and the plasma samples from HC. A PCA 2D scores plot of the normalized and log-transformed data for all samples is shown in Figure 1. Paired *t*-tests were performed, and we identified 792 ions in both positive and negative modes, having a false discovery rate (FDR) <0.05. From the 792 significant ions, some were designated with putative names as well, using MetaboQuest. Ions were further categorized based on the fold change (FC) values in the intensity levels between plasma BC samples and HC samples. A volcano plot showing the significant molecules from the univariate analysis is shown in Figure 2. A fold-change cut-off of 2 was used to select the significant up- and down-regulated molecules.

After adjusting for variables such as the TNM stage, tumor Grade, smoking status, menopausal status, and race (performed by logistic regression with LASSO penalties), 16 ions were selected (12 in the positive electrospray ionization mode and 4 in the negative mode). Their putative identities, based on a mass-based search, is listed in Supplementary Table S1 for the positive mode and Supplementary Table S2 for the negative mode. We observed that some ions were non-metabolites, such as drug-related compounds and/or other dietary supplements. This can be the result of the fact that patients were medicated and undergoing general anesthesia when the blood collection occurred. In Table 3, we list 6 metabolites that have putative identities with potential relevance to breast cancer.

Next, we performed a multivariate analysis (LASSO & SVM-RFE) where a group of 40 BC patients and 40 HC were selected as a training group for the algorithm to obtain a group of classification models, which helped to predict the category of the new data consisting of the remaining 15 BC patients and 15 normal controls (test group). The same analysis was performed for all samples (55 vs. 55) as well. AUC and 95% CI were obtained for each individual metabolite selected by LASSO (Table 4).

**Figure 3:**
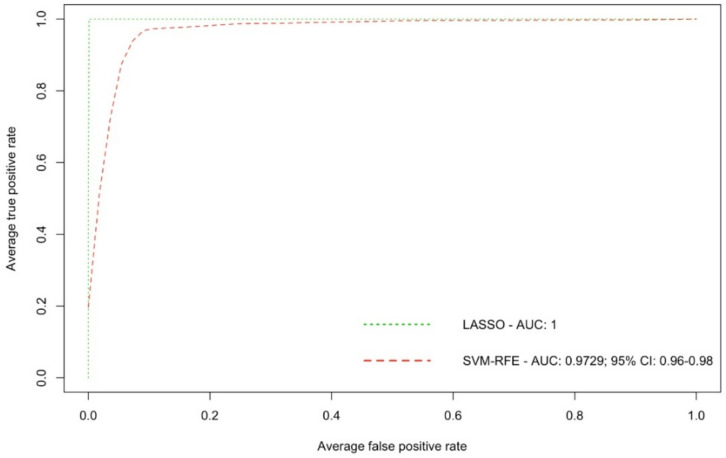
Receiver operating characteristics (ROC) curve with the area under the curve (AUC) showing the prediction performance of the 16 selected ions for all 55 samples.

**Figure 4:**
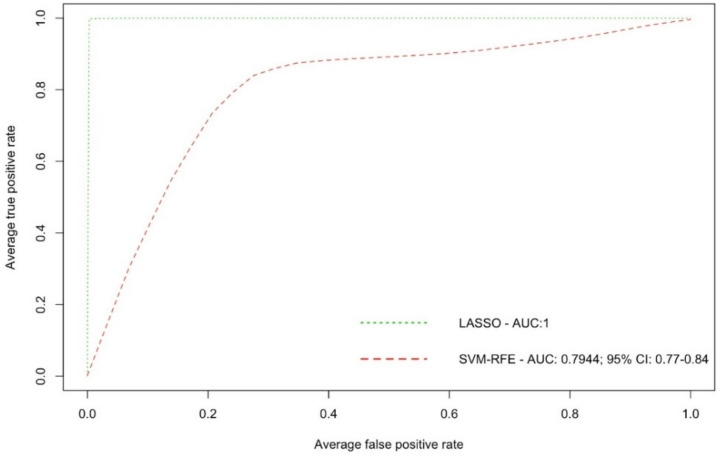
Receiver operating characteristics (ROC) curve with the area under the curve (AUC) showing the prediction performance of the 16 selected ions for the 15 test samples.

We have also investigated the BC biomarker potential for the 16 LASSO-selected ions, to check whether they could be used for BC detection. The diagnostic performance of the 16 ions was evaluated using AUC ROC curves. ROC based on the SVM-RFE granted a very high and accurate diagnostic performance with an AUC value of 0.9729 (95% CI: 0.96–0.98) for all 55 samples (Figure 3). For the 15 test samples, the AUC value was lower (0.7944, 95% CI: 0.77–0.84), yet still indicated a high diagnostic performance (Figure 4).

**Figure 5:**
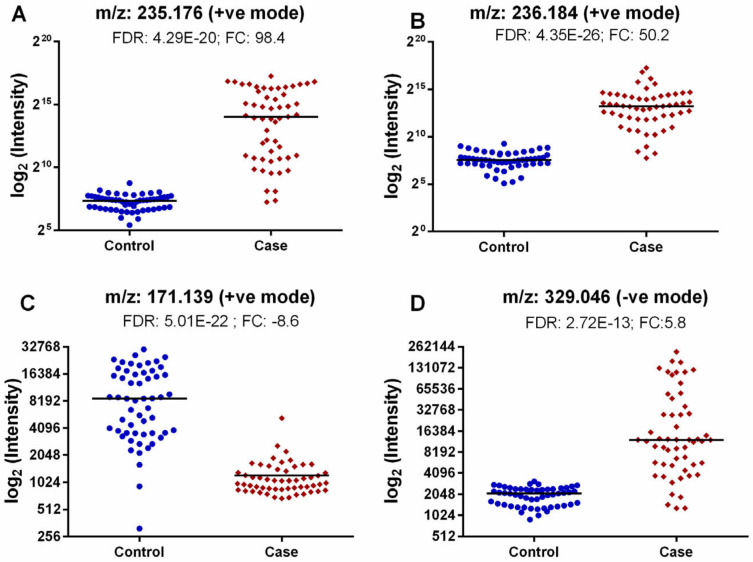
Dot plots of four selected metabolites: L-Arginine (ester), *m*/*z* = 235.176 (**A**); N-stearoyl tryptophan, *m*/*z* = 236.184 (**B**); Caproleic acid, *m*/*z* = 171.139 (**C**); 5-[(4-Nitrobenzoyl)amino]-isophthalic acid, *m*/*z* = 329.046 (**D**).

Most of the metabolites identified were presumably exogenous, originating from either drug metabolism and/or plant-based supplements/essential oils, while some of them were endogenous derivatives of amino acids (N-stearoyl tryptophan, *m*/*z* = 236.184, L-arginine ester, *m*/*z* = 235.176), nitrogenous bases derivatives (Uracil derivative, *m*/*z* = 310.129), or fatty acid/lipid metabolism compounds (Caproleic acid, *m*/*z* = 171.139). We also found one metabolite in the negative ion mode whose spectra matched with the NIST library spectra with a high score, 5-[(4-Nitrobenzoyl)amino]isophthalic acid (329.046). These metabolites are presented in Figure 5.

## 3. Discussion

Similar to genomics, transcriptomics, and proteomics, metabolomics provides critical information on cancer tumorigenesis and progression in various malignancies. The sensitivity of UHPLC/Q-TOF/MS enables researchers to gain insight into the altered metabolic pathways of cancer not only in the tumor microenvironment, but also in blood and other body fluid samples from patients with cancer, as well. Therefore, metabolic profiling can be performed directly on plasma or other liquid biological samples, thus avoiding the need for invasive, unnecessary tissue biopsies.

The aim of our study was to investigate potential differences in the plasma ion intensities of 55 patients diagnosed with BC and 55 healthy controls. We identified 792 ions with significant changes in intensity levels in BC samples vs. HC. Generalized LASSO further selected a total number of 16 ions (12 in the positive ion mode and 4 in the negative ion mode), that presented a high diagnostic performance, having an SVM-RFE AUC value of 0.9729 (95% CI: 0.96–0.98), and therefore could present promising potential as candidate biomarkers for BC detection. We observed significant differences between the plasma samples of BC patients vs. HC at the levels of amino acid derivatives (N-stearoyl tryptophan; increased), nucleic acid derivatives (uracil derivative; decreased), fatty acid biosynthesis, and lipid metabolism-derived compounds (caproleic acid, decreased). Of note, we also observed significant differences for other exogenous molecules, most likely of drug metabolism/dietary supplementation origin, which is consistent with other studies.

Cao et al. (2015) performed a targeted metabolomics analysis on 20 BC patients and 50 healthy volunteers using ESI-QTOF-MS and found elevated levels of tryptophan (*p* < 0.05) in BC patients relative to controls. In addition, attempts were made in order to determine the biological function of tryptophan in its relationship with BC. In vitro studies showed that increased tryptophan levels inhibit IL-10 secretion by CD4+ cells, which could be involved in the pathogenesis of BC [25]. A small-scale study comprising 9 patients with stage II–IV BC was performed in order to investigate the dynamic alpha-[11C]methyl-L-tryptophan (AMT) transport in BC, using positron emission tomography (PET) with tumor immunohistochemistry, and their preliminary data showed that invasive ductal carcinomas had a higher AMT accumulation [26]. Furthermore, previous reports showed a high expression level of indoleamine 2,3-dioxygenase (IDO), known as the rate-limiting enzyme of the kynurenine pathway (responsible for tryptophan oxidation), is found in BC and is correlated with advanced-stage disease. The amino acid exchange transporter LAT1 can also be upregulated in BC, which is correlated with poor prognosis [27,28,29]. What is more, kynurenine showed to be a ligand of the human aryl hydrocarbon receptor (AHR), whose activation promotes cancer development via the suppression of the cellular immune response [30].

Furthermore, we found in our study some metabolites of fatty acid biosynthesis/lipid origin with altered expression levels in BC patients relative to HC. Recent research showed that breast tumors also express a lipogenic phenotype, and the aberrant synthesis of lipid derivatives might drive BC pathogenesis. Both exogenous (dietary intake) and endogenous (de novo lipogenesis) processes are strongly correlated with BC cell proliferation and growth [31,32]. Lipolysis was also found to be upregulated, which is consistent with disease aggressiveness, because monoacyclglyerol lipase (MAGL)-mediated lipolysis contributes to the build-up of the intracellular fatty acid pool, which can be used as the building blocks for the synthesis of lysophospholipids, eicosanoids, and other oncogenic lipid messengers [33]. Furthermore, novel and valid therapeutic targets could arise for the enzymes involved in lipid metabolism, such as inhibitors of lipid messenger signaling, especially because metastatic BC is very responsive to receptor antagonists of small molecular sizes. Successful attempts were achieved in mouse models for the lysophosphatidic acid receptor 1 (LPA1) inhibitor Debio-0719 and LPA1 short hairpinned RNA (shRNA), and for sphingosine-1-phosphate (S1P) signaling. The latter used murine models for treatment with the specific sphingosine kinase 1 (SphK1) inhibitor (SK1-I) which successfully suppressed S1P levels, reduced metastases, and decreased the overall tumor burden [34,35].

Our findings are consistent with the findings of other reported studies, especially in the case of amino acid derivatives (increased tryptophan levels) and fatty acid precursors. Variations in findings across studies could possibly arise due to differences in study designs: sample specimen used (i.e., plasma, tissue, urine, etc.), metabolites extraction protocols, LC-MS methodology, and choice of statistics, in addition to previous treatment received and other exogenous factors (e.g., diet, dietary supplements, etc.). Our study brings considerable novelty to the field of metabolomics-based BC research for the discovery of potential diagnostic biomarkers since this issue has not been investigated using UHPLC/Q-TOF-MS. Suman et al. (2018) previously conducted a similar study comparing metabolic signatures of early- and late-stage BC using ^1^H NMR spectroscopy-based metabolomics, which is known to have a lower sensitivity and a decreased number of detectable metabolites per analysis. However, their reported results, although different from ours, contained molecules from similar classes (i.e., amino acids, fatty acids, and/or lipid metabolism-derived products) [36,37]. In addition, to further refine and increase the confidence of our results, we have selected significant metabolites only following LASSO regression after the adjustment of several variables (TNM stage, BC grade, smoking status, menopausal status, and race), which has never been done in studies with a similar design and aims. Other reported studies have investigated, to a great extent, the metabolic signatures of BC, mainly to discover the altered signaling pathways and further explain the differences in metabolites concentrations between BC samples and controls; however, little evidence exists regarding the usefulness of these metabolites as diagnostic biomarkers for this malignancy, and therefore, our study could open novel avenues into the future analysis of plasma-derived molecules as a minimally-invasive tool for the early detection of BC.

Limitations of our study include the fact that our sample size (55 BC patients and 55 HC) was relatively small, which makes it difficult to determine whether these metabolites could be useful in future BC screening and characterization. Secondly, our results should be viewed as preliminary, since we have used an untargeted approach in our study design, encompassing all metabolites from plasma samples of BC patients and HC. Lastly, our ROC analysis pooled dietary/exogenous compounds together with endogenous molecules, introducing, therefore, potential heterogeneity to the accurate interpretation of the results. The vast majority of the altered metabolites that we have discovered were of an exogenous origin, mostly derived from drug catabolism. This might be explained by the fact that the blood samples from our BC patients were collected under anesthesia immediately before surgery when the patient was already medicated. In addition, some of the patients were undergoing therapy, preoperatively. However, the differences in intensity levels for the drug-related compounds could bring resourceful insights for future BC therapy (i.e., enzyme targeting, pharmacokinetic analysis, etc.).

Taken together, these data grant promising confidence that the novel era of metabolomics-based biomarkers for BC detection could represent a non-invasive, early disease-identification approach, that could complement common strategies used at present, with optimized performances. As improvements in understanding BC metabolism are substantially growing, future approaches will require independent validation in large-scale powered studies with an appropriate selection of patients, in order to increase the confidence of the findings and advance to the stepping-stone era of metabolomics-based profiling for BC tumor characterization and diagnosis. The eventual integration of the metabolomic data with other omics data from the same patients, i.e., genomics, transcriptomics, and proteomics, will definitely improve the characterization of BC and its early detection.

## 4. Materials and Methods

### 4.1. Patient Population and Plasma Samples

Our retrospective case-control study included a total of 110 subjects: fifty-five patients with biopsy-confirmed, invasive BC, and/or ductal carcinoma in situ (DCIS), and 55 age- and race-matched healthy controls (HC). BC patients were scheduled for surgery in the Department of Surgery at MedStar Georgetown University Hospital, Washington DC, USA, and the blood was collected under anesthesia immediately before surgery. Data regarding age, menopausal status, race, smoking history, histopathological examination, BC stage, BC grade and status of estrogen, progesterone, and HER-2 receptor were extracted by the physician from patients’ medical files. All subjects signed an IRB-approved informed consent for the use of their biological samples prior to blood collection, and their samples had been de-identified in downstream analysis. This study was approved by the Georgetown University Institutional Review Board (IRB). Venous blood was drawn in EDTA-coated collection tubes, centrifuged (15 min, 2000× *g*) for the separation of plasma, and plasma aliquots were immediately frozen at −80 °C for further use.

### 4.2. Metabolite Extraction from Plasma and LC-MS Analysis

A 25 µL aliquot of plasma was combined with 75 µL of 40% isopropanol, 25% methanol, and 35% water containing the internal standards debrisoquine and 4-benzoic acid. The samples were vortexed and left on ice for 20 min then 100 µL of acetonitrile was added. The samples were then left at −20 °C for 20 min, and then were centrifuged at 15,493× *g* for 20 min at 4 °C. The supernatant for each sample was taken for LC-MS analysis. The samples were injected onto a Waters reverse phase BEH C18 column (Waters Corp., Milford, MA, USA). The mobile phase consisted of water containing 0.1% formic acid (solvent A), acetonitrile containing 0.1% formic acid (solvent B), and 90% isopropanol and 10% acetonitrile containing formic acid (solvent C). The flow rate was set to 0.4 mL/min and each sample was resolved over a 13-min window. The gradient was set as follows: initial—97% A, 3% B; 0.5 min—97% A, 3% B; 4.0 min—40% A, 60% B; 8.0 min—2% A, 98% B; 9.0 min—5% B, 95% C; 10.0 min—5% B, 95% C; 11 min—25% A, 25% B, 50% C; 13 min—97% A, 3% B. The samples were analyzed using a Waters G2 quadrupole time-of-flight mass spectrometer with a 50 to 1200 *m*/*z* range. Electrospray ionization operating in either positive or negative ionization mode was used to introduce the column eluent into the mass spectrometer. The positive mode capillary voltage was set to 1.6 kV and the negative mode capillary voltage was set to 1.5 kV. Both ionization modes had a cone voltage of 30 V, a desolvation gas flow of 600 L/h, a cone gas flow of 25 L/h, and a source temperature of 120 °C. Data were acquired in the sensitivity mode and had a scan time of 0.3 s with an interscan delay of 0.0014 s. Leucine Enkephalin (556.2771 [M + H] +/554.2615 [M − H]−) at a concentration of 1.0 ng/mL was infused via the Waters Lockspray interface every 10 s to apply real time mass correction.

### 4.3. Data Pre-Processing and Statistical Analysis

Data were obtained from plasma samples of 110 subjects (55 with BC and 55 HC), in both electrospray positive and negative ion modes. The data were pre-processed using R-package XCMS (Ver 2019, Scripps Center for Metabolomics, La Jolla, CA, USA) after being converted into Network Common Data Format (NetCDF) using the MassLynx software (Waters Corp., USA), as previously described [22]. R-package CAMERA was subsequently used, following peak matching using XCMS, to identify potential adducts, isotopes, and in-source fragments, as indicated [38]. Prior to statistical analysis, clustering of similar elution profiles was performed using CAMERA.

Univariate and multivariate statistical analyses were performed to pre-screen the ions. Paired *t*-tests followed by FDR correction were performed for the profile comparisons of metabolites between cases (BC patients) and healthy subjects. Fold changes (FC) and *p*-values were obtained and calculated using MetaboAnalyst. In addition, generalized LASSO regression model from the R package (‘glmnet’) was used to further discriminate ions associated with BC/normal plasma samples. Parameters such as TNM stage, BC grade, smoking status, menopausal status, and race were adjusted for. Conditional logistic (LASSO penalty-based) regression was applied using ‘clogitL1’. The diagnostic value for the selected ions were evaluated using the receiver operating characteristic (ROC) curves and by calculating the area-under-the-curve values.

Finally, metabolites were identified with putative names (10 ppm mass tolerance) using MetaboQuest, a cloud-based tool that searches for putative metabolite IDs in major compound databases based on *m*/*z* values (http://tools.omicscraft.com/MetaboQuest/, accessed on 9 May 2022).

**Figure 6:**
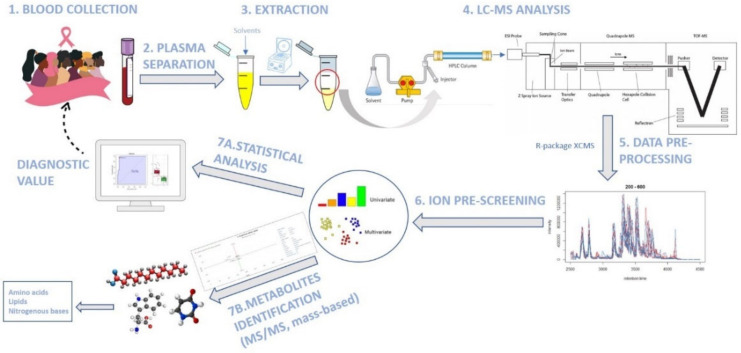
Summarized steps of the Materials and Methods sections.

A brief scheme with all methodological steps can be seen in Figure 6.

## Figures and Tables

**Table 1:** Characteristics of the BC patients (N = 55) included in our study.

Characteristics	N = 55	% *
**Age (mean ± SD)**	53.35 (±12.26)	
**Menopausal status**		
Pre-menopause	18	32.73%
Peri-menopause	2	3.64%
Post-menopause	35	63.64%
**Race**		
Asian	5	9.09%
African American	15	27.27%
Caucasian	33	60.00%
Hispanic	2	3.64%
**Smoking history**		
Current smoker	7	12.73%
Former smoker	5	9.09%
Never smoked	43	78.18%
**Histology**		
Ductal carcinoma in situ (DCIS)	10	18.18%
Invasive ductal carcinoma (IDC)	35	63.64%
Invasive lobular carcinoma (ILC)	5	9.09%
Mixed	2	3.63%
**BC stage**		
0	9	16.36%
I	21	38.18%
II	15	27.27%
III	4	7.27%
**BC grade**		
Low	7	12.72%
Intermediate	21	38.18%
High	25	45.45%
**Lymph node involvement**		
Yes	14	25.45%
No	40	72.72%
**Estrogen receptor (ER)**		
Positive	45	81.82%
Negative	9	16.36%
**Progesterone receptor (PR)**		
Positive	33	60.00%
Negative	21	38.18%
**HER2 status**		
Positive	11	20.00%
Negative	39	70.90%
**Operative procedure**		
Bilateral mastectomy (BM)	18	32.73%
Preventive mastectomy (PM)	14	25.45%
Mastectomy	22	40.00%
Endoscopy-assisted breast surgery (EABS)	1	1.82%
**Palpable tumor**		
Yes	25	45.45%
No	30	54.55%

* Might not add to 100% due to missing data.

**Table 2:** The total number of ions detected and ions selected by statistical analysis.

Ion Mode	Number of Ions Detected	Number of Ions with Adjusted *p* Value < 0.05
Positive (POS)	1930	603
Negative (NEG)	564	189

**Table 3:** Metabolites selected with putative identities.

Metabolite ID	Formula	*m*/*z*	Exact Mass	Ion Mode	Retention Time (RT)	BC vs. HC	Fold Change (FC)	Adjusted *p* Value
**Caproleic acid**	C10H18O2	171.139	170.131 (M + H)	+	314.469	↓	−8.62	1.00 × 10^−24^
L-Arginine (ester) *	C8H18N4O2	235.176	202.143 (M + CH3OH + H)	+	150.534	↑	98.43	1.21 × 10^−22^
**N-stearoyl tryptophan**	C29H46N2O3	236.184	470.354 (M + 2H)	+	135.323	↑	50.15	5.23 × 10^−29^
Ile-Ser *	C9H18N2O4	236.184	426.386 (M + 2Na)	+	135.323	↑	50.15	5.23 × 10^−29^
**Uracil (derivative)**	C15H20ClN3O2	310.129	309.124 (M + H)	+	55.740	↓	−17.82	6.85 × 10^−24^
**Met-His-OH**	C16H18N4O6S	395.103	394.095 (M + H)	+	361.000	↑	2.06	1.64 × 10^−11^
5-[(4-Nitrobenzoyl)amino]isophthalic acid *	C15H10N2O7	329.046	330.049 (M − H)	−	323.114	↑	5.84	2.72 × 10^−13^

* These metabolites have been identified using matched MS/MS identifications (Supplementary Figures S1–S3). The other identifications were done via mass-based putative identifications from public databases.

**Table 4:** Multivariate analysis (LASSO & SVM-RFE) on training, test, and all samples.

Metabolite		Train Samples (40 vs. 40)		Test Samples (15 vs. 15)		All Samples (55 vs. 55)	
*m*/*z*	Ion Mode	AUC	95% CI AUC	AUC	95% CI AUC	AUC	95% CI AUC
171.139	+	0.971	0.922–1	0.916	0.787–1	0.969	0.931–1
203.107	+	0.925	0.864–0.985	0.809	0.620–0.997	0.904	0.843–0.964
221.118	+	0.970	0.939–1	0.844	0.666–1	0.940	0.886–0.992
223.064	+	0.884	0.801–0.967	0.920	0.788–1	0.911	0.850–0.971
235.176	+	0.968	0.925–1	1.000	1	0.976	0.944–1
236.184	+	0.974	0.941–1	1.000	1	0.980	0.954–1
256.942	+	0.746	0.638–0.853	0.987	0.957–1	0.681	0.581–0.780
302.122	+	0.940	0.881–0.998	0.893	0.774–1	0.929	0.877–0.981
306.977	+	0.913	0.842–0.983	0.711	0.501–0.920	0.875	0.803–0.947
310.129	+	0.951	0.883–1	0.858	0.720–0.995	0.937	0.882–0.990
395.103	+	0.855	0.764–0.945	0.769	0.572–0.965	0.842	0.761–0.922
451.165	+	0.898	0.831–0.963	0.764	0.580–0.948	0.876	0.810–0.940
223.028	−	0.897	0.817–0.975	0.907	0.772–1	0.912	0.851–0.972
317.948	−	0.949	0.893–1	0.667	0.451–0.881	0.889	0.819–0.959
329.046	−	0.988	0.971–1	0.836	0.657–1	0.953	0.907–0.998

## Data Availability

Not applicable.

## Supplementary Materials

The following supporting information can be downloaded at: https://www.mdpi.com/article/10.3390/metabo12050447/s1, Figure S1: Matched spectra for L-arginine ethyl ester; Figure S2: Matched spectra for 5-[(4-Nitrobenzoyl)amino]isophtalic acid; Figure S3: Matched spectra for Ile-Ser; Table S1: Putative identities, based on a mass-based search, for the positive ion mode; Table S2: Putative identities, based on a mass-based search, for the negative ion mode.
