# Supplementary figures and images for: Metabolomic Analysis of Plasma from Breast Cancer Patients Using Ultra-High-Performance Liquid Chromatography Coupled with Mass Spectrometry: An Untargeted Study
Source: Metabolites. 2022 May 17;12(5):447. doi: 10.3390/metabo12050447 (PMC9147455; doi:10.3390/metabo12050447)

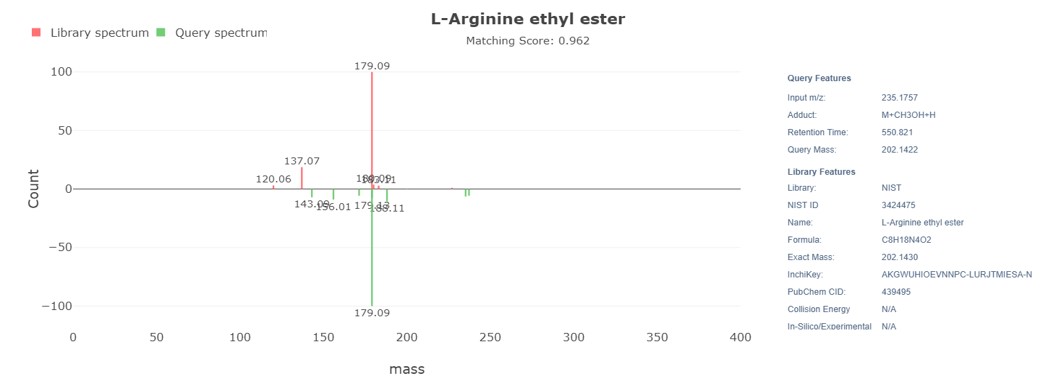

Supplement: Supplementary file 1 [file metabolites-12-00447-s001.zip › Figure S1.jpg]

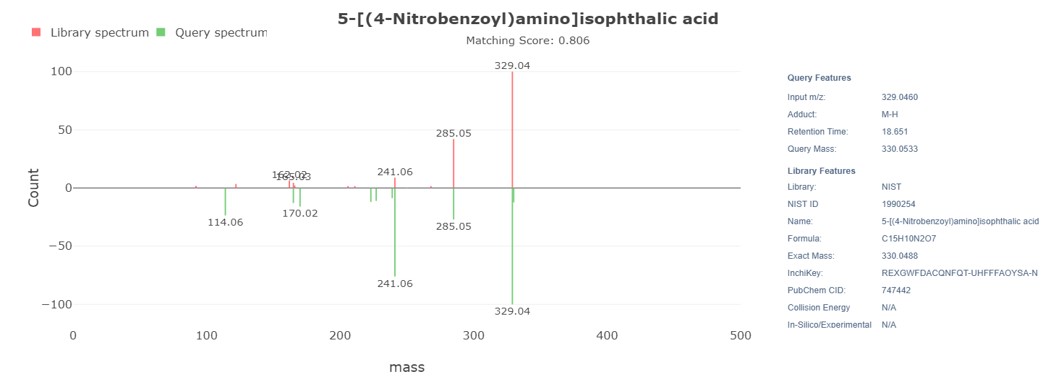

Supplement: Supplementary file 1 [file metabolites-12-00447-s001.zip › Figure S2.jpg]

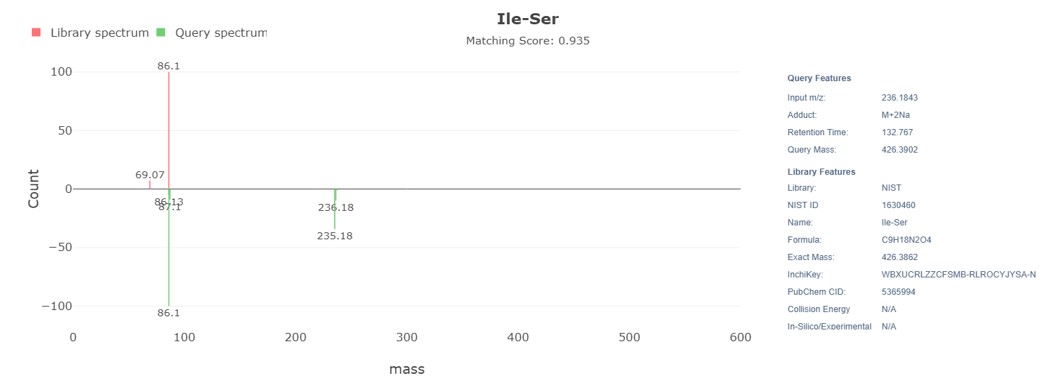

Supplement: Supplementary file 1 [file metabolites-12-00447-s001.zip › Figure S3.jpg]
